# Mesenchymal Stem Cells Do Not Lose Direct Labels Including Iron Oxide Nanoparticles and DFO-^89^Zr Chelates through Secretion of Extracellular Vesicles

**DOI:** 10.3390/membranes11070484

**Published:** 2021-06-29

**Authors:** Yue Gao, Anna Jablonska, Chengyan Chu, Piotr Walczak, Miroslaw Janowski

**Affiliations:** 1 Department of Diagnostic Radiology and Nuclear Medicine, University of Maryland School of Medicine, Baltimore, MD 21201, USA; Yue.Gao@som.umaryland.edu (Y.G.); ajablonska@som.umaryland.edu (A.J.); chengyan.chu@som.umaryland.edu (C.C.); pwalczak@som.umaryland.edu (P.W.); 2 Department of Neurointervention, Dalian Municipal Central Hospital Affiliated with Dalian Medical University, Dalian 116033, China; 3 Department of Neurology, Dalian Municipal Central Hospital Affiliated with Dalian Medical University, Dalian 116033, China

**Keywords:** extracellular vesicles, labeling, stem cells, cell tracking, imaging

## Abstract

Rapidly ageing populations are beset by tissue wear and damage. Stem cell-based regenerative medicine is considered a solution. Years of research point to two important aspects: (1) the use of cellular imaging to achieve sufficient precision of therapeutic intervention, and the fact that (2) many therapeutic actions are executed through extracellular vesicles (EV), released by stem cells. Therefore, there is an urgent need to interrogate cellular labels in the context of EV release. We studied clinically applicable cellular labels: superparamagnetic iron oxide nanoparticles (SPION), and radionuclide detectable by two main imaging modalities: MRI and PET. We have demonstrated effective stem cell labeling using both labels. Then, we obtained EVs from cell cultures and tested for the presence of cellular labels. We did not find either magnetic or radioactive labels in EVs. Therefore, we report that stem cells do not lose labels in released EVs, which indicates the reliability of stem cell magnetic and radioactive labeling, and that there is no interference of labels with EV content. In conclusion, we observed that direct cellular labeling seems to be an attractive approach to monitoring stem cell delivery, and that, importantly, labels neither locate in EVs nor affect their basic properties.

## 1. Introduction

Stem cell-based regenerative medicine is expected to address the growing problem of tissue wear and damage in rapidly ageing populations [1,2]. Mesenchymal stem cells (MSCs) are one of the most versatile therapeutic candidates [3,4]. With the advent of precision medicine, attention is increasingly being paid to the accuracy of cell transplants and the ability to track the administration of cells to confirm their location to the target tissue. There are a variety of cellular labels, although few have strong clinical potential. Magnetic and radioactive labels are the most prevalent due to their very high sensitivity and detection by magnetic resonance imaging (MRI) and positron emission tomography (PET), both clinically applicable imaging modalities [5,6]. While studies on the stability of radioactive labeling of stem cells are scarce, there are a lot of confusing data with regard to magnetic labeling of stem cells, which is worth further investigation. This is especially true because many therapeutic activities of MSCs seem to be mediated by the extracellular vesicles (EVs) released by them [7].

EVs are cell-released nanoparticles comprised mainly of proteins, lipids, and nucleic acids [8,9]. There is growing evidence that EVs play a vital role in mediating intercellular communication [10]. However, there is a lack of well-controlled studies which examine whether direct cell labeling affects the process of EV production, and if the cellular labels are passed on in released EVs. This has important implications related to the reliability of labels to report on the transplanted cells, or if labels are released and taken up by other cells, thereby confusing the interpretation of cell tracking. Another possible repercussion is related to the impact of cellular labels on the process of EV production, perhaps deteriorating their functionality by replacing potentially therapeutic cargo with neutral labels.

Therefore, in this study, we compared the production of EVs by labeled cells and naïve cells to investigate the passing of cellular labels to EVs.

## 2. Materials and Methods

### 2.1. Cell Culture

Human bone marrow mesenchymal stem cells (hMSCs) (passage 1) were purchased from Rooster Bio (RoosterVial™-hBM-10M-XF, Frederick, MD, USA). They were cultured in T75 flasks at 2 × 10^5^ cells with 10 mL of Rooster Nourish-MSC-XF xeno-free culture media at 37 °C in a humidified atmosphere containing 5% CO_2_. The medium was changed every three days, and cells were passaged after reaching 70–80% confluency. The cells from up to 5 passages were used for experiments.

### 2.2. Magnetic Labeling of HMSCs

The hMSCs cells were labeled with Molday ION superparamagnetic iron oxide nanoparticles (SPIO), size 35 nm and charge ~+31 mV, purchased from BioPAL (Worcester, MA, USA). The labeling procedure included the incubation of 70–80% confluent hMSCs in Rooster Nourish-MSC-XF medium supplemented with Molday ION at a 20 μg/mL concentration for 16 h (overnight).

### 2.3. EV Isolation

EVs were isolated from labeled and control 70–80% confluent hMSCs. EV isolation was preceded with hMSC suspension with 10 mL of RoosterCollect-EV cell culture. Initially, hMSCs were incubated with RoosterCollect-EV medium for two hours to remove all the serum, and subsequently cultured in the new RoosterCollect-EV medium for 48 h. After that time, cell culture supernatants were collected and centrifuged for 5 min at 1300× *g* to remove cells and debris, followed by filtering through a 0.45 μm syringe filter to remove all large particles. Then the EVs were isolated and concentrated with ultrafiltration on 300 kDa centrifugal filtering columns Vivaspin (Sartorius, Germany). The EVs were washed twice with 0.22 μm membrane-filtered phosphate-buffered saline (PBS) to allow the full exchange of the cell culture medium to PBS. The collected EVs were stored at −20 °C until further analyzed.

### 2.4. Tunable Resistive Pulse Sensing (TRPS)

The size distribution, concentration, and zeta potential of EVs derived from labeled and control cells were analyzed with tunable resistive pulse sensing (tRPS) technology using the qNano Gold system (Izon Science Ltd., Christchurch, New Zealand). The NP150 nanopores (analysis range 70–420 nm; Izon Science) were used in this study for EVs analysis. Before measurement, the nanopore was wetted and coated according to the manufacturer’s instructions. The voltage, stretch, and pressure were first adjusted on the CPC 220 calibration beads (Izon Science, Christchurch, New Zealand) so that the relative blockade magnitude ranged from 0.25%–4% and the particle speed was within 10–15/ms. The same parameters were then applied for measuring EV samples. The beads and samples were analyzed until 500 particles were counted. Data processing and analysis were performed using the Izon Control Suite software v3.3 (Christchurch, New Zealand).

### 2.5. Flow Cytometry

The isolated EVs were stained with vFluor Red Membrane Stain according to manufacturer protocol (The vFCTM Vesicle Analysis Kit; (Cellarcus, San Diego, CA, USA). Briefly, the membrane stain solution was incubated with EVs for 1 h at RT in the dark. After that time, dilution (30×) of stained sample in staining buffer was made, and samples were run on CytoFlex (Beckman Coulter, Brea, CA, USA) at 60 µL/min flow rate. As a negative control, the Lipo100 Standard was used. To determine presence of PE labelled Molday ION particles in the isolated EVs, the unstained fraction of EVs was suspended in PBS and run at the same setting on CytoFlex (Beckman Coulter, Brea, CA, USA).

### 2.6. In Vivo MR Imaging of EVs Derived from Molday ION-Labeled HMSCs

All surgical procedures were approved by The University of Maryland Animal Care and Use Committee. A C57BL/6 mouse (6–8 weeks old) was used for in vivo EV imaging to further validate the success of Molday ION labeling (Biopal, Worcester, MA, USA). Under general anesthesia, intra-carotid catheterization was performed as we described previously [11]. The animal was then transferred to a Bruker 9.4 T MRI scanner. Baseline T2 (TR/TE = 2500/30 ms) and dynamic gradient echo-echo planar imaging (GE-EPI, TR/TE 1250/9.7 ms, field of view = 14, matrix = 128, acquisition time = 60 s and 24 repetitions) images of the brain were acquired. GE-EPI MRI enables visualization of the infused MR contrast agent. The dissolved Molday ION (Biopal, Worcester, MA, USA) (1:30, 0.3 mg Fe/mL) was first infused via the intra-arterial catheter at a rate of 0.15 mL/min under dynamic GE-EPI MRI to highlight the perfusion territory and to serve as a positive control. The EVs derived from Molday ION-labeled hMSCs were thereafter infused using the same catheter.

### 2.7. ^89^Zr Labelling of Cells for EVs Production

The ^89^Zr oxalate was mixed with 1M HEPES buffer, pH was adjusted to 7 and 5 mM, and p-SCN-Bn-Deferoxamine (DFO) was added. The mix was incubated for 1 h in 37 °C mixed at 550 RPM. Chelation efficiency was determinate by silica gel iTLC, showing 99% efficacy of ^89^Zr-DFO complex creation. The media in 80% confluent T75 flask of hMSCs was changed to one with decreased concertation of FBS (2% FBS), and ^89^Zr-DFO complexes were added directly to the media (200 and 400 µCi). Cells were incubated with radioactive complexes overnight. Next, cells were washed twice with DMEM to remove unbound ^89^Zr, and fresh DMEM was added for production of EVs. After 2 days, media were collected and EVs were isolated using an ultrafiltration system. The ^89^Zr activity was measured in supernatant, cells and EVs fractions.

### 2.8. Image Processing and Statistical Analysis

MRI imaging was processed using Image J, and the signal change was plotted with GraphPad 8.0. PROC MIXED (SAS 9.4) was used for statistical analysis, with the lowest means square (LMS) test used to compare groups. The statements “repeated” and “random” were used for repeated measures and to express random effects.

## 3. Results

### 3.1. HMSCs Labeling with Molday ION

**Figure 1:**
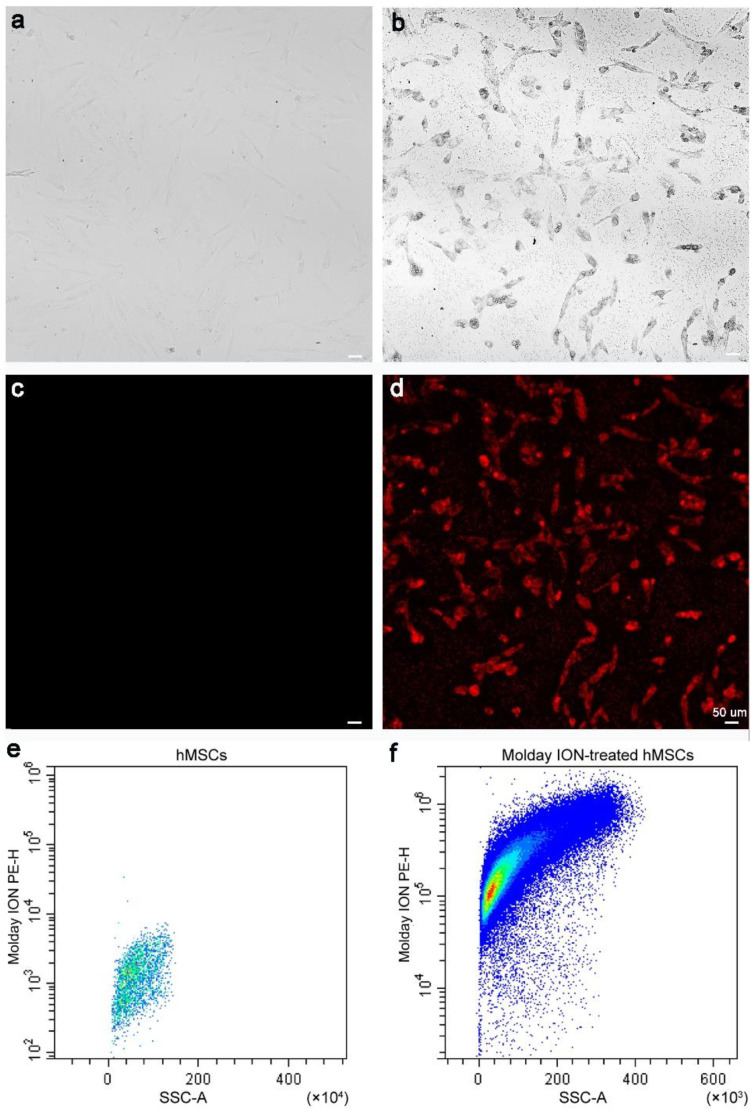
Microscopic and flow cytometry analysis of labeled hMSCs: Bright-field images of non-labeled (**a**) and Molday ION-treated hMSCs (**b**), fluorescent microscopy also of non-labeled (**c**) and Molday ION-treated hMSCs (**d**), and flow cytometry analysis of hMSCs of non-labeled (**e**) and Molday ION-treated hMSCs (**f**).

The hMSCs were observed under a bright-field microscope to determine their Molday ION uptake. In the Molday ION-treated cells, the presence of SPIO was determined by visualization of black spots in the cytoplasm (Figure 1a), which are not visualized in naïve hMSCs (Figure 1b). Under a fluorescence microscope, red fluorescence was found in hMSCs treated with Molday ION labeled with Rhodamine B, which is not observed in naïve hMSCs (Figure 1c,d). Additionally, in Molday-treated hMSCs, flow cytometry showed detectable Molday ION signal (Molday ION labeled with Rhodamine B) but was absent in naïve hMSCs (Figure 1e,f). All the data confirmed the success of labeling hMSCs with Molday ION.

### 3.2. The Effect of Molday ION Treatment on the Number and Size of HMSCs-Derived EVs

**Figure 2:**
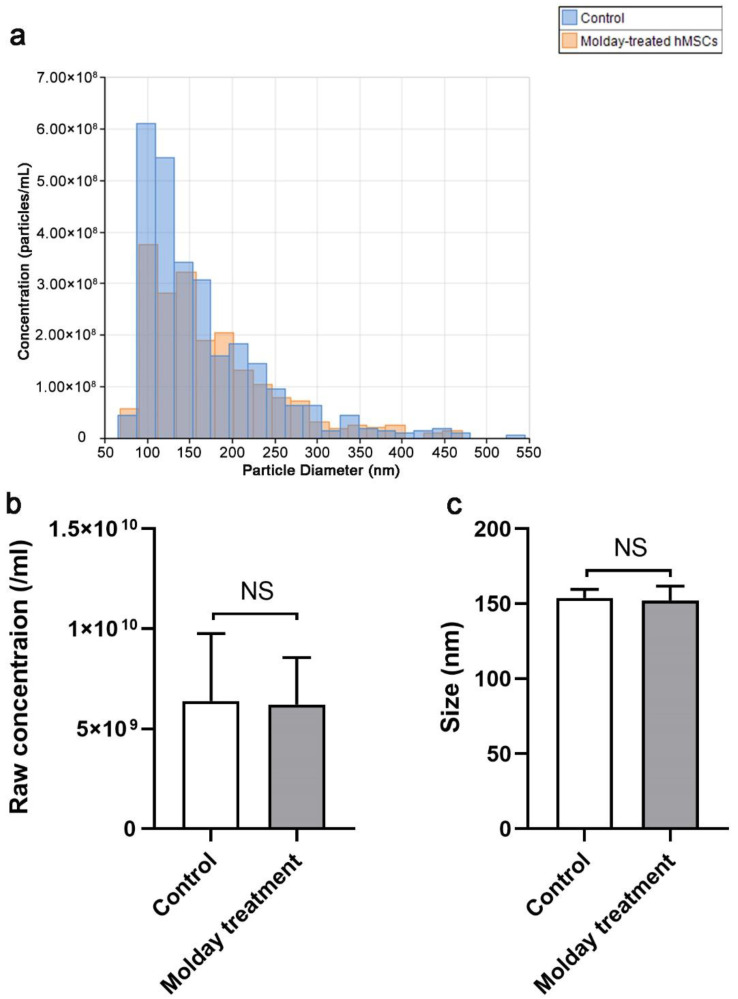
Size distribution, concentration, and size comparison of EVs derived from Molday ION-treated and control hMSC: (**a**) Size distribution of EVs derived from Molday ION-treated and control hMSC. (**b**,**c**) Concentration and size comparison of EVs derived from Molday ION-treated and control hMSC. NS: No significant difference between treatment groups.

We did not observe any differences in the size distribution of EVs derived from hMSCs labeled with Molday ION and control cells (Figure 2a). Additionally, we also counted the EV particles from the same density of hMSCs and Molday ION-treated hMSCs. We did not find any significant difference in EVs’ number and size between these two groups (Figure 2b). Overall, our data revealed that Molday ION treatment of hMSCs does not affect the production and size of the EVs produced by them.

### 3.3. The Effect of Molday ION Treatment on the Zeta Potential of HMSCs-Derived EVs

**Figure 3:**
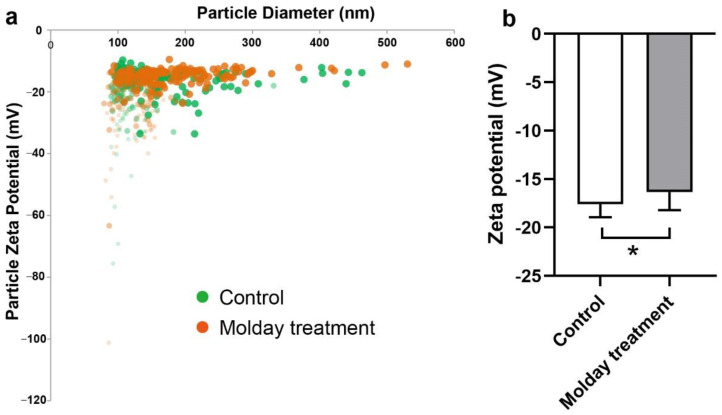
Zeta potential comparison of control hMSC-EVs and Molday ION-treated hMSC-EVs: (**a**) Zeta potential distribution of EVs derived from control and Molday ION-treated hMSCs. (**b**) Zeta potential comparison of hMSC-EV control group (n = 6) and Molday ION treated hMSC-EV group (n = 8). * Significant difference between treatment groups.

The effect of Molday treatment on EVs’ potential was also examined using the tRPS system. The potential distribution of EVs from hMSCs and Molday treated hMSCs was first profiled. We found that the EVs had a similar distribution (Figure 3a). The average zeta potential was then calculated. As a result, the average potential of EVs from control hMSCs was slightly higher at −16.3 mV than that from Molday ION-treated hMSCs at -17.6 mV (Figure 3b). The statistical analysis determined a significant difference between them, indicating that Molday ION is somewhat increasing the negative charge of hMSCs-derived EVs.

### 3.4. Characterization of HMSCs-Derived EVs and the Efficacy of Labeling EVs with Molday ION

**Figure 4:**
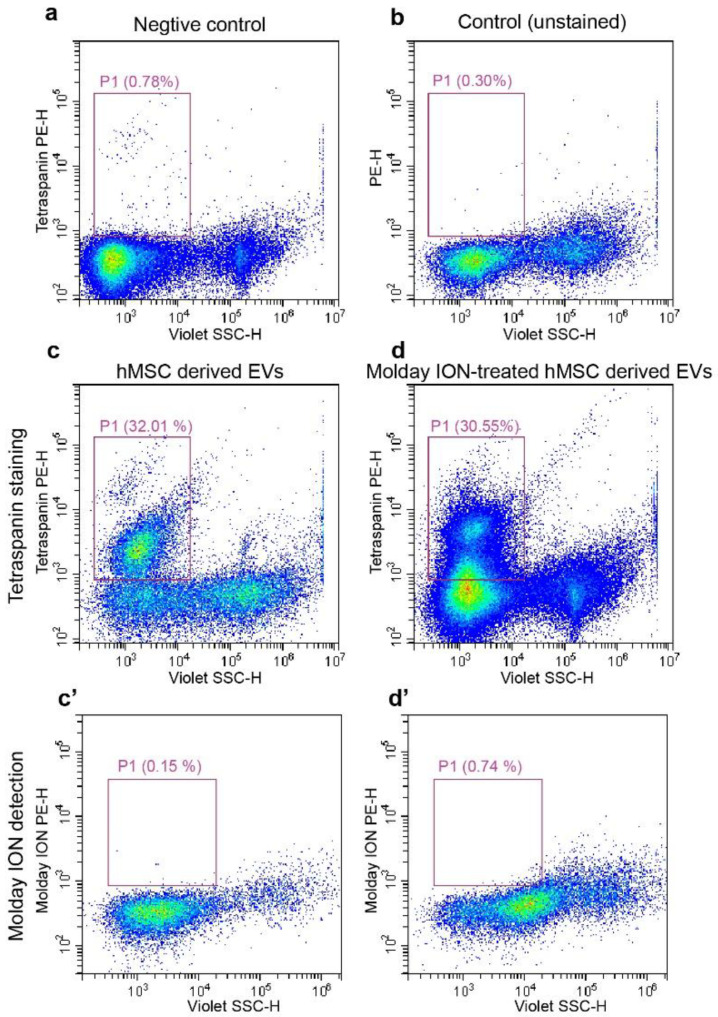
Flow cytometry analysis on isolated EVs: Liposomes stained with tetraspanin and unstained EVs isolated from hMSCs were used as negative control (**a**) and regular control (**b**), respectively. The representative flow cytometry images of tetraspanin positive for EVs from control (**c**), and Molday ION-treated hMSCs (**d**). Molday ION detection in EVs from untreated (**c’**) and Molday-treated hMSCs (**d’**).

Flow cytometry was performed using antibodies against tetraspanins as a specific stain for EVs. We first examined the specificity of antibodies through incubation with liposomes. Flow cytometry showed that liposomes were negative for tetraspanins (Figure 4a). The hMSC-EVs and Molday ION-treated hMSC derived EVs were then found to be similarly positive for tetraspanins (38.11% ± 8.11% and 36.01% ± 5.41%, respectively) in contrast to those in the unstained control (0.23% ± 0.07%) (Figure 4a–d). To further validate the specificity of tetraspanin staining, the tetraspanins-stained EVs were incubated with Triton X to destroy EV vesicles. Subsequently, the second flow cytometry analysis showed no tetraspanin-positive EVs (data not shown). Thus, our data indicated the specificity of staining for MSC-EVs, thereby confirming their exosomal property.

It was proved that hMSCs were labeled with Molday; this does not imply that Molday ION was packed into the EVs. Flow cytometry was then used to verify the success of Molday labeling for EVs characterized in Figure 4c,d. Unexpectedly, the subsequent flow cytometry analysis showed a lack of Molday ION signal in both experimental and control EV populations (Figure 4c’,d’), indicating a lack of passing labels from MSCs to EVs secreted from them.

### 3.5. In Vivo MR Imaging of EVs Derived from Molday ION-Treated HMSCs

**Figure 5:**
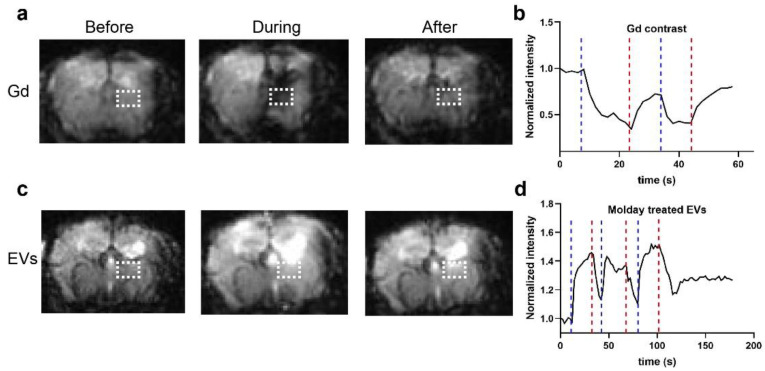
Real-time MRI to observe the EV delivery: (**a**) Representative T2* images before, during, and after infusion of SPIO at a rate of 0.15 mL/min. (**b**) Dynamic signal changes of the two ROIs marked in (**a**). (**c**) Representative T2* images before, during, and after infusion of EVs from Molday ION-treated hMSCs at a rate of 0.15 mL/min. (**d**) Dynamic signal changes of the two ROIs marked in (**c**).

While flow cytometry gave a definitive response, it analyzed a fluorescent molecule attached to SPIONs. Here, we used in vivo MRI to ultimately draw conclusions regarding the useful presence of SPIONs in the EVs. As we reported previously, the infusion of SPION-based contrast results in a signal drop (hypointensity) on T2* MRI, which immediately disappears after the injection [11]. Such hypointensity in the brain can be sampled by GE-EPI MRI, enabling visualization of the contrast perfusion in the brain parenchyma in real time. We first injected Molday ION at a rate of 0.15 mL/min via the intra-arterial catheter, and Molday ION transcatheter perfusion territory appeared in the brain (Figure 5a), as visualized by a remarkable reduction in signal intensity for the duration of the injection bolus (Figure 5b). Afterward, the EVs from Molday ION-treated hMSCs were infused using the same catheter; there was no visible hypointensity perfusion area in the brain (Figure 5c). The signal intensity for the duration of the injection bolus also confirmed the absence of contrast agent perfusion, as no signal reduction was observed (Figure 5d). In comparison, the signal even went up slightly during the EVs injection (Figure 5d). Altogether, the data further imply that the SPIONs are not passed from Molday ION-treated hMSCs to EVs derived from them.

### 3.6. The Passing of ^89^Zr-DFO-Based Radiolabel from MSCs to EVs Derived from Them

**Figure 6:**
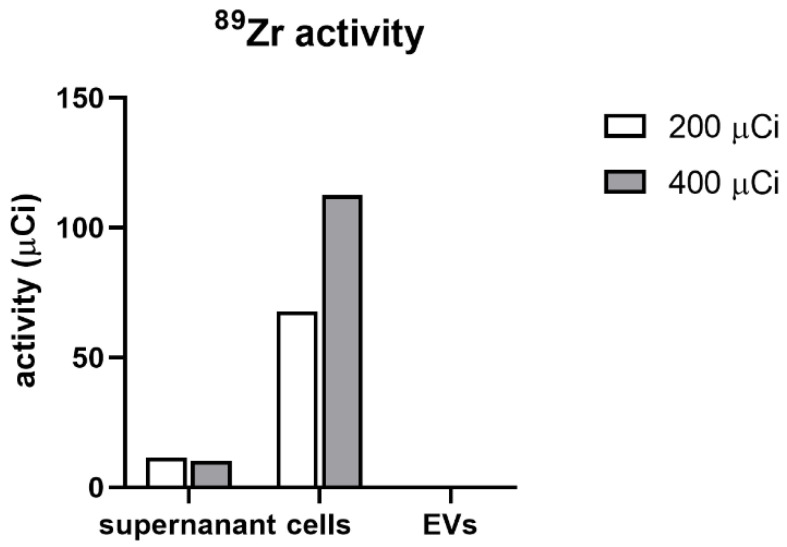
The activity of ^89^Zr in the supernatant, cells and isolated EVs.

After 2 days of EV production, the supernatant, cells, and isolated EVs were analyzed for activity of ^89^Zr (Figure 6). We observed that both the experimental variants (200 and 400 µCi) and the cells were successfully labeled with the activity of 67.7 and 112.6 µCi, respectively. Moreover, in both experimental flasks, we observed some ^89^Zr activity present in cell media, 11.57 µCi for 200 µCi flask and 10.35 µCi for the 400 µCi flask. After isolation of EVs with ultrafiltration in both tested doses, the activity in this fraction was minimal, with 0.4 µCi in 200 µCi and 0.6 µCi in 400 µCi group. Therefore, we conclude that neither of the tested radioactivity doses lead to passing ^89^Zr-DFO radiolabel from MSCs to EVs derived from them.

## 4. Discussion

We have shown that frequently used cellular labels, magnetic SPIONs and ^89^Zr-DFO complexes, do not pass from parent hMSCs to EVs derived from them. We have also demonstrated that SPION-based labeling does not affect the number and size of EVs, and has minimal effect on their charge.

These results are important because they address recent inquiries in the literature. Early studies revealed a very interesting phenomenon in which transplanted SPION-labeled neural stem cells (NSCs) stayed in place, while migrating NSCs were missing their label. This may indicate a massive SPION efflux from NSCs through EVs, or asymmetric NSC division with SPION restriction to one sedentary cell and another SPION-free motile cell [12]. A subsequent in vitro study failed to show such asymmetric NSC divisions [13]. Therefore, there was a high probability of massive release of SPION through EVs, which could indicate a characteristic of EVs released by MSCs. In this context, our data provides strong support for the idea that the losing of SPION is not a highly generalizable phenomenon as it was not observed in our experiments, which is highly encouraging. Thus, our experiments demonstrated that SPION can be used for stable MSC labeling. We have also demonstrated that not only relatively large nanoparticles, such as SPIONs, are not passed on EVs, but also very small radioactive tracers, such as ^89^Zr-DFO chelates, which are also absent in EVs obtained from radiolabeled MSCs. Moreover, we have shown that magnetic labeling of MSCs does not affect EV production in terms of the number of EVs, their size, as well as the presence of tetraspanins on their surface, which is also very positive information for the potential of cellular labeling and therapeutic transplantation of transplanted cells. This is important, as we have previously demonstrated that SPION has negligible impact on the MSCs’ stemness characteristic and their other properties in vitro [14]. Therefore, it seems that direct labels are quite resistant to packaging them to the EVs, so there is minimal chance that cell labeling will diminish EV-mediated positive therapeutic consequences of MSC transplantations. However, this information is not fully generalizable and similar studies should be performed prior to proposing new direct cellular labels.

Notably, the current study has different implications related to the administration of EVs themselves as therapeutic agents. The possibility of imaging and monitoring EV administration is equally important in the case of stem cells in order to better understand their therapeutic potential and the fate of EVs, as well as to accelerate the translation of extracellular vesicle-based therapies [15]. Thus, a reliable method for in vivo noninvasive assessment of administrated EVs is highly desirable. MRI has been widely used in preclinical and clinical studies due to its multiple advantages, including its radiation-free, high spatial resolution. Magnetic agents such SPION agents have been approved in Europe for treatment of anemia and commercially available for human use, but also are frequently used off-label for MR imaging. In fact, SPION has been used to directly label EVs by electroporation for longitudinal tracking and visualization. In a mouse study, Hu et al. succeeded in labeling melanoma-derived EVs with SPION, and the labeled EVs were tracked using MRI for up to 48 h after injection [16]. A recent study reported by Han et al. loaded SPION-coated with polyhistidine tags into pluripotent stem cell (iPSC)-derived EVs, which were shown to preferentially accumulate in the injury sites and conferred substantial protection [17]. The high efficacy of direct EV radiolabeling also has been reported recently [18]. However, both studies included a procedure of direct labeling, which adds another step after EV isolation and may affect EV characteristics [19].

Therefore, it would be very convenient to label cells and enable them to pass labels to EVs derived by them. No additional step of EV labeling would be needed, which would simplify EV processing and preparation for in vivo administration. Two studies proved the feasibility of this concept. In a study reported by Busato et al. adipose stem cells (ASC) were labeled with SPION and they found that the collected EVs were tagged [20]. This was then confirmed by another study using SPION and MSCs [21]. Our study was more geared toward therapeutic applications, so we used Rooster Nourish-MSC-Xeno-Free culture media as well as a different method of EV isolation, which is likely to yield much better purified EVs, thus eliminating contamination by freely available labels. There are also no xeno-sourced raw materials in the formulation, thereby circumventing any remaining safety issues related to xeno-sourced animal components for clinical translation [22,23,24].

The current study has several limitations, such as a lack of data from electron microscopy and Western blots. We would like to emphasize the extraordinary circumstances of the COVID-19 pandemic, which interfered with our plans. In addition, we would like to point out that we were using a very precise TRPS method to count and provide basic characterization at the level of single EVs, which is by far more advantageous than optical methods used by the majority of scientists, which allow only to average EV properties. Moreover, the data from flow cytometry are robust and clear-cut with all necessary controls, thus adding Western blot would not change our message.

In conclusion, in the present study we have shown that the secreted EVs do not carry the labels (SPION, ^89^Zr-DFO) from their parent cells. Moreover, the labels do not seem to affect EV production. These findings assure the value of direct cell labeling for subsequent in vivo tracking. However, a caution for further studies regarding labeling EVs using this indirect approach needs to be placed, and probably direct EV labeling needs to be embraced.

## Data Availability

The data presented in this study are available on request from the corresponding author.
